# Exploring the role of oral microorganisms in the pathogenesis of mucositis by assessing their impact on metabolic activity and reproductive capacity of epithelial cells in vitro

**DOI:** 10.1007/s00520-020-05318-y

**Published:** 2020-01-22

**Authors:** Thijs M. Haverman, Alexa M. G. A. Laheij, Min Nie, Dong M. Deng, Judith E. Raber-Durlacher, Johannes J. de Soet, Frederik R. Rozema

**Affiliations:** 1 Department of Oral Medicine, Academic Centre for Dentistry Amsterdam (ACTA), University of Amsterdam and the Vrije Universiteit Amsterdam, Gustav Mahlerlaan 3004, 1081 LA Amsterdam, The Netherlands; 2 Department of Preventive Dentistry, Academic Centre for Dentistry Amsterdam, University of Amsterdam and the Vrije Universiteit Amsterdam, Amsterdam, The Netherlands; 3 State Key Laboratory of Oral Diseases, West China Hospital of Stomatology, Sichuan University, Chengdu, China; 4 Department of Oral- and Maxillofacial Surgery, Amsterdam UMC, University of Amsterdam, Amsterdam, The Netherlands

**Keywords:** Oral mucositis, Oral epithelial cells, Wound healing, *P. gingivalis*, *Candida* spp., Clonogenic assay

## Abstract

**Purpose:**

Clinical and in vitro studies showed selected oral microorganisms to be related to delayed wound healing and ulcerative oral mucositis. However, it is not known whether this effect is due to reduced metabolism and/or the reduced reproductive capacity of epithelial cells. Therefore, we studied the influence of the oral microorganisms *Porphyromonas gingivalis*, *Candida glabrata*, and *Candida kefyr* on cell metabolism and reproductive capacity of oral epithelial cells, aimed to further unravel the pathogenesis of oral mucositis.

**Methods:**

Oral epithelial cells were exposed to different concentrations of *P. gingivalis*, *C. glabrata*, and *C. kefyr* as mono-infections or mixed together. An MTT assay was performed to determine the effect on cell metabolism. A clonogenic assay was used to study the effect on the reproductive capacity of oral epithelial cells.

**Results:**

The metabolism of oral epithelial cells was reduced when the microorganisms were present in high concentrations: *P. gingivalis* at a multiplicity of infection (MOI) of 1000 and the *Candida* spp. at MOI 100. No statistical difference was observed in the ability of a single epithelial cell to grow into a colony of cells between control and *P. gingivalis*, *C. glabrata*, and *C. kefyr*, independent of the concentrations and combinations used.

**Conclusion:**

*P. gingivalis*, *C. glabrata*, and *C. kefyr* lowered the metabolic activity of oral epithelial cells in high concentrations, yet they did not influence the reproductive capacity of epithelial cells. Their impact on ulcerative oral mucositis is likely due to an effect on the migration, proliferation, and metabolism of epithelial cells.

## Introduction

Cancer patients undergoing radiotherapy to the head and neck area or receiving high dose chemotherapy often suffer from severe oral mucositis [23]. Ulcerative oral mucositis is characterized by the loss of mucosal integrity and is associated with opioid use, dietary problems, weight loss, higher healthcare costs, and local and systemic infections [7, 20, 23]. The underlying pathogenesis of oral mucositis is described in a five-phase model involving initiation, upregulation and generation of messenger signals, signal amplification, ulceration, and healing [3, 20, 21].

There is increasing evidence that microorganisms play a crucial role in the ulceration and healing phases of oral mucositis, thereby contributing to its severity and duration [19, 21, 22]. It was found that the gram-negative anaerobic oral bacterium *Porphyromonas gingivalis* and the oral yeasts *Candida glabrata* and *Candida kefyr* were associated with the presence of oral ulcerations in hematopoietic stem cell recipients [12].

*P. gingivalis* possesses well-known virulence factors, such as LPS, gingipains, and fimbriae, that help the bacterium in surviving. *P. gingivalis* is able to invade epithelial cells and to remain internalized, probably due to the production of proteolytic enzymes, like gingipains [2]. Others also identified internalization of gingipains as important contributors to cellular impairment [6, 14].

Since healing of ulcerations is important in the pathogenesis of oral inflammatory diseases including mucositis, in vitro wound healing models are used to study the influence of oral microorganisms on this process. It was demonstrated that *P. gingivalis*, *C. glabrata*, and *C. kefyr* strongly inhibited wound closure in vitro [1, 8, 10, 13].

Besides hindering the migration of oral epithelial cells, *P. gingivalis* causes cellular impairment by invading epithelial cells and producing proteolytic enzymes like gingipains [2, 6, 15]. Moreover, *P. gingivalis* is associated with enhanced apoptosis and decreased proliferation of oral epithelial cells in vitro [1, 2, 26]. In high concentrations, *Candida albicans* induces apoptosis of epithelial cells during the early stages of infection [24, 25].

The short-term effects of *P. gingivalis* and *C. albicans* on several wound healing processes in oral epithelial cells were studied before. Long-term effects, however, did not receive much attention in literature. An option for studying long-term effects is the clonogenic assay [5]. This is an in vitro assay based on the ability of a single cell to grow out into a colony, which stands for the reproductive capacity of cells. This validated assay studies possible DNA damage in human cells that may result in less cells after several cell divisions. The aim of this study was to assess the influence of the non-hyphae-forming yeasts *C. glabrata*, *C. kefyr*, and oral pathobiont *P. gingivalis* on the metabolic activity and the reproductive capacity of oral epithelial cells in the context of wound healing in vitro.

## Materials and methods

### Epithelial cells

The oral epithelial cell line CA9-22 was used. The cells were grown in an incubator at 37 °C in a humidified atmosphere containing 5% CO_2_ and 95% air within Dulbecco’s modified Eagle’s medium Ham’s F-12 nutrient mixture (DMEM-F12; Invitrogen, Carlsbad, CA, USA), completed with 10% fetal calf serum (Hyclone, Logan, UT), 100 IU/mL of penicillin, 100 μg/ mL of streptomycin, and 250 ng/mL of amphotericin B (all from Sigma–Aldrich, St. Louis, MO, USA). After confluency was reached (Corning, New York, NY, USA), the cells were detached using 0.25% trypsine-EDTA (Invitrogen) and counted with a hematocytometer.

### Bacterial strains and culture

*P. gingivalis* ATCC 33277 was cultured anaerobically (80% N_2_, 10% H_2_, and 10% CO_2_) in brain-heart infusion (BHI; 37 g/L;BD Difco, Le Pont de Claix, France) supplemented with hemin (5 mg/L) and menadione (1 mg/L). *C. glabrata* CBS 138 and *C. kefyr* CBS 1970 were cultured aerobically at 37 °C in amino acid-depleted, glucose-enriched yeast nitrogen base (YNB; 6.7 g/L; BD Difco). All microorganisms were grown until log phase, which was ascertained by measuring the optical density (OD_690_ for *P. gingivalis* and OD_600_ for the *Candida* spp.). Subsequently, the microorganisms were washed twice with Dulbecco’s phosphate-buffered saline (DPBS; Gibco, NYC, USA), re-suspended in keratinocyte serum-free medium (SFM; Gibco, NYC, USA), and subsequently diluted until the desired concentration. The relationship between the colony-forming units and corresponding ODs was determined beforehand. For each clonogenic assay, a freshly prepared bacterial and/or yeast culture was used. All cultures were checked for purity and hyphal growth by culturing and gram staining. All cultures were pure and no hyphal growth was observed.

### Metabolic activity

In order to assess the activity of oral epithelial cells after co-incubation with *P. gingivalis* and *Candida* spp., an MTT (3-(4,5-dimethyl-2-thiazolyl)-2,5-diphenyl-2-H-tetrazolium bromide) assay was carried out. This assay measures the ability of cells to reduce the tetrazolium dye MTT to an insoluble purple formazan by succinate dehydrogenase within their mitochondria [4]. Since this reaction requires functional mitochondria, it effectively measures the metabolic activity of living cells.

The oral epithelial cells were seeded in Dulbecco’s minimum essential medium (DMEM, Sigma, St.Louis, USA) in a 96-well plate at a concentration of 8 × 10^4^ per well and incubated for 16 h in a CO_2_ incubator (5% CO_2_) at 37 °C. After removal of the medium, the cells were rinsed with 200 μl DPBS (Gibco, NY, USA). Viable *P. gingivalis*, *C. glabrata*, and *C. kefyr* were added to the wells, for each concentration in triplicate, and incubated for 4 h in the CO_2_ incubator. For both *Candida* spp., a multiplicity of infection (MOI) of 100, 10, and 1 was used; for *P. gingivalis*, an MOI of 1000, 100, and 10 was used. In control wells, SFM only was added as a negative control. After the removal of the microorganisms, the epithelial cells were rinsed twice with DPBS. Then 100 μl 0.5 mg/ml MTT solution (3-(4,5-dimethyl-2-thiazolyl)-2,5-diphenyl-2-H-tetrazolium bromide) was added to each well and incubated for 2 h in the CO_2_ incubator. Finally, the MTT solutions were removed, 100 μl dimethyl sulfoxide (DMSO, Sigma, St. Louis, USA) was added to each well in order to dissolve the insoluble formazan, and the optical density of DMSO in each well was measured at 570 nm.

### Clonogenic assay

The clonogenic assay was performed according to the protocol of Franken et al. [5]. Briefly, in each well of a 6-well plate, 250 epithelial cells were seeded in 3 ml DMEM-F12 medium with fetal calf serum, without antibiotics and antimycotics. After attachment, the epithelial cells were challenged with viable *P. gingivalis*, *C. glabrata*, and *C. kefyr* (prepared as described above) with a corresponding MOI of 10, 1, and 0.1 for the *Candida* spp. and 100, 10, and 1 for *P. gingivalis*. The control group was treated with SFM only (Gibco, NYC, USA).

After 17 h, the medium with microorganisms was removed and the epithelial cells were washed with DPBS. Subsequently, DMEM-F12 with fetal calf serum, penicillin, streptomycin, and amphotericin B was added to the wells. The plates were then placed in a CO_2_ incubator (5% CO_2_) at 37 °C and left there for a time equivalent to at least six potential cell divisions (approximately 7 days). During that period, the medium was refreshed three times.

On day 7, the medium was removed and the epithelial cells were carefully rinsed with DPBS. Then the epithelial cells were treated with a mixture of 6.0% glutaraldehyde and 0.5% crystal violet to fix and stain the cells. After at least 30 min, this mixture was removed and the cells were carefully rinsed with tap water twice. Finally, the plates with the epithelial cells were dried in normal air at room temperature and the colonies were counted using a stereomicroscope. One colony was defined as a cluster of at least 50 cells [5]. Each treatment was performed in triplicate, and each experiment was performed on at least three separate occasions.

The survival fraction was calculated. First, the plating efficiency per well was determined. The plating efficiency is the ratio of the number of colonies that were formed after 7 days divided by the number of cells seeded [5]. The epithelial cells were seeded in two plates, and each two plates had its own control wells. The number of cells that were seeded was equal for the control and treated wells. Subsequently, the relative survival fraction was determined by dividing the plating efficiency from the challenged cells by the average plating efficiency of the control wells. In other words, the relative survival fraction is the fraction of treated cells that grew into a colony versus the numbers of cells seeded, relative to the untreated cells.

Relative survival fraction =$\frac{\mathrm{No}.\mathrm{of}\ \mathrm{colonies}\ \mathrm{formed}}{\mathrm{No}.\mathrm{of}\ \mathrm{cells}\ \mathrm{seeded}}\times 100\%$/average plating efficiency control. The relative survival fraction under control (untreated) conditions was 1. If the relative survival fraction was smaller than 1, less epithelial cells had survived relative to the control cells. If greater than 1, more epithelial cells had survived relative to the control cells.

### Statistical analysis

The Kruskal-Wallis test was used to determine differences in reproductive capacity of the epithelial cells under different microbial conditions compared to control. The Mann-Whitney *U* test was used to calculate any differences in metabolic activity of the epithelial cells under different microbial conditions compared to control. Statistical analyses were performed with IBM SPSS Statistics for Windows v25 (IBM Corp., Armonk, NY, USA). A *p* value of < 0.05 was considered to be significantly different.

## Results

### Metabolic activity

**Fig. 1:**
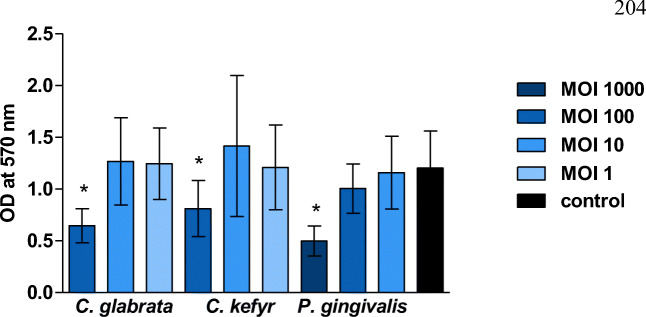
Mean optical density (± SD) in wells with oral epithelial cells exposed to different concentrations of viable *C. glabrata*, *C. kefyr*, or *P. gingivalis*. Significant differences compared to control are marked with an asterisk

The metabolic activity of oral epithelial cells exposed to different concentrations of viable *C. glabrata*, *C. kefyr*, or *P. gingivalis* is shown in Fig. 1. When the three microorganisms were present in high concentrations, the metabolic activity of oral epithelial cells was lower than that of the control group. For *C. glabrata* and *C. kefyr*, this was the case for MOI 100 (*p* = 0.002 and 0.030, respectively), and for *P. gingivalis,* this was the case for MOI 1000 (*p* < 0.05). All other concentrations of the mono-infections had no effect on the metabolic activity compared to control.

**Fig. 2:**
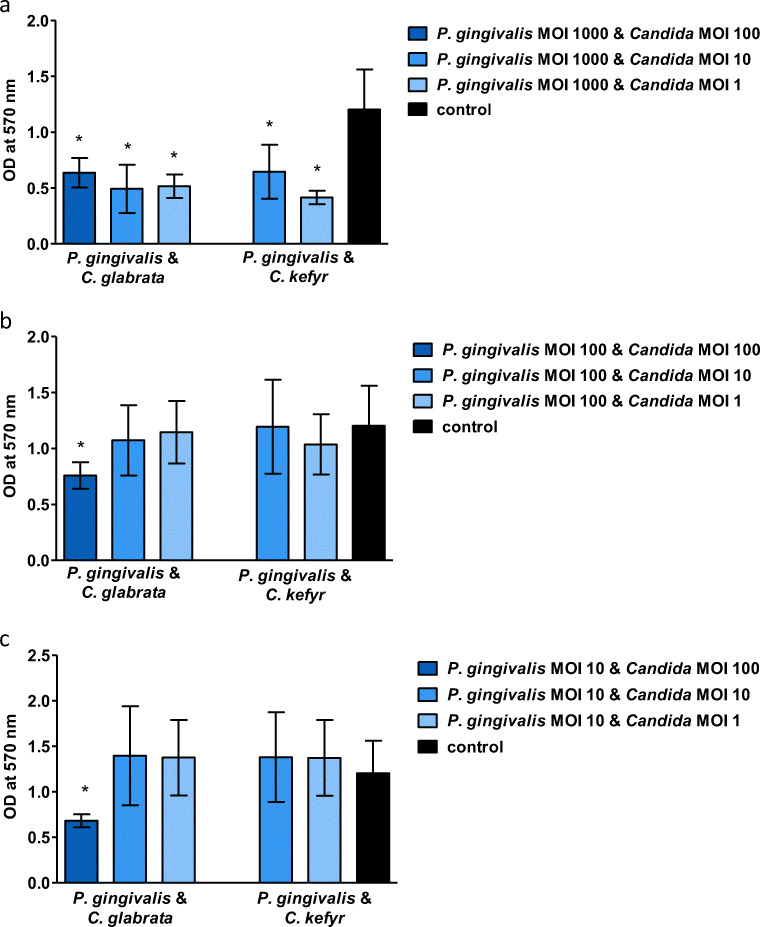
Mean optical density (± SD) in wells with oral epithelial cells exposed to different concentrations and combinations of viable *C. glabrata* or *C. kefyr* and *P. gingivalis*. Significant differences compared to control are marked with an asterisk

The same trend was observed in experiments with the mixed infections (see Fig. 2). If *C. glabrata* or *P. gingivalis* were present at MOI 100 and 1000, respectively, the metabolic activity of the oral epithelial cells was significantly lower compared to control (*p* < 0.05). No additive effect was observed of a mixed infection compared to a mono-infection. It turned out not to be possible to prepare a mixed infection with a high concentration of *C. kefyr* (MOI 100), since the viscosity of the suspension was too high to handle and therefore no results could be obtained.

### Clonogenic assay

**Fig. 3:**
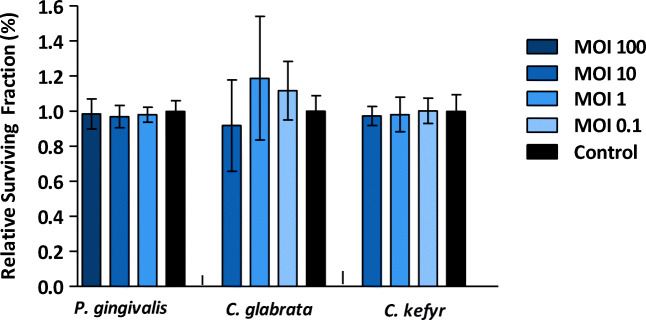
Mean relative survival fraction (± SD) of oral epithelial cells exposed to different concentrations of viable *P. gingivalis*, *C. glabrata*, and *C. kefyr*

**Fig. 4:**
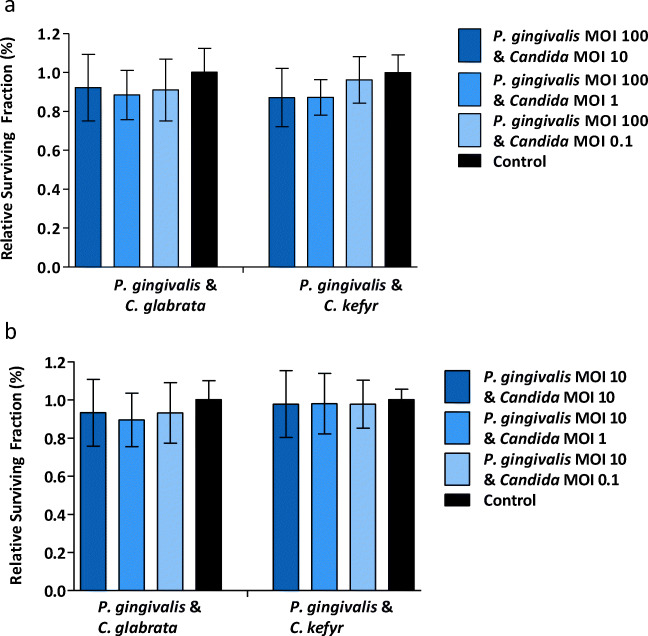
Mean relative survival fraction (± SD) of oral epithelial cells exposed to different concentrations and combinations of viable *C. glabrata* or *C. kefyr* with *P. gingivalis*

The relative survival fraction of the oral epithelial cells challenged with viable *C. glabrata*, *C. kefyr*, or *P. gingivalis* as a mono infection compared to control is shown in Fig. 3. No significant difference was observed between the relative survival fraction of epithelial cells in the control group and epithelial cells after exposure to different concentrations of *C. glabrata* and *C. kefyr* (*p* = 0.214 and *p* = 0.925, respectively). And no difference was found between different concentrations of *P. gingivalis* and control (*p* = 0.762). The relative survival fraction of epithelial cells exposed to a mixed infection of *C. glabrata* or *C. kefyr* and *P. gingivalis* is shown in Fig. 4. Again, no significant differences were observed between groups, independent of the concentrations and combinations used. These results mean that the ability of oral epithelial cells to undergo unlimited cell division was not hindered by *P. gingivalis*, *C. glabrata*, or *C. kefyr*.

## Discussion

Since the oral microorganisms *C. glabrata*, *C. kefyr*, and *P. gingivalis* are related to ulcerative oral mucositis and delayed wound healing, the aim of this study was to look into the influence of these microorganisms on the metabolic activity and the reproductive capacity of oral epithelial cells. The highest concentrations of the microorganisms studied lowered the metabolic activity of these cells. The effect of *P. gingivalis* on metabolic activity of oral epithelial cell was studied before, but slightly different models were used, for instance, primary oral epithelial cells vs cell lines, short (2–6 h) vs longer exposure times (24–48 h), and different MOIs (10–1000) [2, 11, 16, 26]. It seems that a short exposure and lower concentrations of *P. gingivalis* had no effect on the metabolic activity of oral epithelial cells, while longer exposure times and high concentrations lowered the metabolic activity, which is consistent with our results. It was also shown before that exposure to a high concentration of *Candida albicans* lowered the metabolic activity of epithelial cells [17].

Studies on the influence of oral microorganisms on oral cells in the context of wound healing have mostly focused on its short-term elements including proliferation, migration, and signaling pathways or cell cycles, and not the reproductive capacity of cells, which is a long-term element in wound healing [6, 11, 15, 16]. Yet, the reproductive capacity of cells seems to be another important property of cells that may be linked to wound healing. The clonogenic assay was introduced in 1956 by Puck and Marcus [18]. It is an in vitro cell survival assay based on the ability of a single cell to grow into a colony [5]. The essence of such an assay is to determine the ability to undergo “unlimited” cell division and the reproductive capacity. If a certain treatment results in damage of the genetic material, this assay is able to detect the cells that have, despite the damage, preserved their capacity for cell reproduction. The ability to proliferate continuously is essential for tissue integrity and function [5]. Although the assay is originally designed to study the effect of irradiation, others successfully used this assay to study the effect of microorganisms on the reproductive capacity of cells [9, 27].

Interestingly, in our study, *C. glabrata*, *C. kefyr*, and *P. gingivalis* had no effect on the reproductive capacity of the oral epithelial cells. The cells were still able to undergo unlimited cell division after a challenge with the different concentrations of these microorganisms either as a mono-infection or in combination with each other. We found the enzymatic activity of the epithelial cells to be inhibited. However, this was not a strong and long-lasting effect, potentially since damage to the genetic material of the oral epithelial cells was repaired and the epithelial cells preserved their ability to divide unlimitedly.

Microorganisms such as the soil bacterium *Streptomyces puniceus* and the vaginal protozoan *Trichomonas vaginalis* had a negative effect on the reproductive ability of epithelial cells [9, 27]. While the microorganisms we studied proved to impede migration and proliferation of oral epithelial cells in a dose-dependent way [2, 8, 13], it is unlikely that this inhibition of wound healing was caused by damage to the ability of oral epithelial cells to undergo unlimited cell division after exposure to these microorganisms.

*C. glabrata*, *C. kefyr*, and *P. gingivalis* were associated with oral mucositis in hematopoietic stem cell transplant recipients [12] and delayed wound healing in vitro [8, 13]. Several short-term elements of wound healing, such as proliferation, migration, and metabolic activity, were proven to be negatively affected by *P. gingivalis* [2, 26]. In the present in vitro study, we found that *P. gingivalis*, *C. glabrata*, and C. *kefyr* lowered the metabolic activity of oral epithelial cells in high concentrations; however, they did not influence the reproductive capacity of these cells. Their role in ulcerative mucositis is likely due to effects on early components of wound healing such as decreasing migration, proliferation, and cell metabolism of epithelial cells.
